# Platelet-rich plasma in the management of musculoskeletal disorders: a narrative review of clinical evidence and practical considerations

**DOI:** 10.3389/fmed.2026.1920226

**Published:** 2026-09-15

**Authors:** Stavros Litsos, Andreas P. Diamantopoulos, Marius Molund

**Affiliations:** 1 House of Health, Oslo, Norway; 2 Rheumatology, Akershus University Hospital, Lørenskog, Norway; 3 Sykehuset Østfold HF, Grålum, Norway

**Keywords:** intra-articular injection, musculoskeletal, osteoarthritis, platelet-rich plasma, PRP, regenerative medicine, tendinopathy

## Abstract

**Background:**

Platelet-rich plasma (PRP) has emerged as a widely applied autologous biologic therapy in musculoskeletal medicine. Its concentrated growth factor content is postulated to promote tissue regeneration, modulate the inflammatory cascade, and provide clinically meaningful and durable symptom relief in both degenerative joint disease and chronic tendinopathy. Despite its widespread clinical adoption, the evidence base remains heterogeneous, and its rational application requires a critical synthesis of current findings.

**Objective:**

This narrative review aimed to critically examine the existing clinical evidence for PRP in the management of degenerative joint disorders and soft tissue conditions, to evaluate indication-specific efficacy, and to provide evidence-based clinical recommendations for its use in musculoskeletal practice.

**Methods:**

A narrative review of the literature was undertaken, with searches of Medline (via PubMed), Embase, and the Cochrane Database of Systematic Reviews for relevant publications up to July 2025. Randomized controlled trials (RCTs), systematic reviews, and meta-analyses comparing PRP with active comparators or placebo in osteoarthritis or tendinopathy were selected based on clinical relevance, sample size, and follow-up duration. No formal protocol registration, dual independent screening, or risk-of-bias scoring was applied; the synthesis is therefore interpretive rather than systematic.

**Results:**

PRP may provide clinically meaningful symptom improvement in selected patients with mild-to-moderate knee and hip osteoarthritis, where modest advantages over hyaluronic acid are reported in some studies and medium-term outcomes may be more durable than with corticosteroid, particularly with leukocyte-poor PRP (LP-PRP) delivered as a series of two to three injections. Among soft tissue conditions, PRP appears most useful relative to corticosteroid in plantar fasciitis, degenerative rotator cuff disease, and trochanteric tendinopathy. Evidence in lateral epicondylitis is mixed: PRP outperforms corticosteroid in the medium term, but placebo-controlled data do not demonstrate a clinically important effect. Current evidence does not support PRP for Achilles tendinopathy, and placebo-controlled data also challenge its use in ankle osteoarthritis. The therapeutic effect of PRP is characteristically delayed, emerging at eight to sixteen weeks and persisting up to eighteen to twenty-four months in selected indications; however, the certainty of evidence varies substantially by indication, comparator, PRP formulation, and follow-up duration.

**Conclusion:**

PRP may represent a reasonable second-line treatment option for selected musculoskeletal conditions, particularly after insufficient response to structured conservative management. The strength of evidence is highly indication-dependent and is weakest precisely where placebo-controlled data exist. Standardization of PRP preparation and reporting, and rigorous placebo-controlled trials in indications where these are lacking, are essential to advance the evidence base.

## 1 Introduction

Musculoskeletal disorders encompass a broad spectrum of conditions affecting joints, tendons, and periarticular soft tissues, representing a principal cause of chronic pain, functional disability, and reduced health-related quality of life worldwide. Conventional management strategies—including pharmacological treatment, physiotherapy, and corticosteroid injection—provide symptomatic relief for many patients; however, a significant proportion experiences insufficient, transient, or progressively diminishing benefit, particularly in the context of chronic tendinopathy and degenerative joint disease. Surgical intervention, whilst effective in advanced cases, is associated with inherent procedural risks and prolonged rehabilitation, and is not appropriate for all patient groups. These therapeutic limitations have stimulated growing interest in biologically active regenerative treatments capable of addressing the underlying pathophysiology rather than merely suppressing symptoms.

Platelet-rich plasma (PRP) has emerged as one of the most extensively studied autologous biologic interventions in the musculoskeletal field over the past two decades (1–4). The treatment is predicated on the concentration of autologous platelets—obtained by centrifugation of the patient's own whole blood—and their subsequent re-injection at the site of pathology. Upon activation, platelets release a repertoire of bioactive growth factors, including platelet-derived growth factor (PDGF), transforming growth factor-beta (TGF-β), vascular endothelial growth factor (VEGF), insulin-like growth factor-1 (IGF-1), and epidermal growth factor (EGF). These mediators are proposed to influence fibroblast and chondrocyte activity, angiogenesis, and extracellular matrix remodeling, although the translation of these biological effects into clinically meaningful tissue repair remains uncertain (1, 5–7).

In contrast to corticosteroids, which suppress inflammation non-selectively and may compromise tissue integrity with repeated use, PRP has been proposed to exert more tissue-directed immunomodulatory and anabolic effects, including modulation of pro-inflammatory cytokines such as interleukin-1 beta (IL-1β) and tumor necrosis factor-alpha (TNF-α) (1). In tendinous tissue, PRP may influence tenocyte metabolic activity and collagen remodeling, although definitive evidence of restored tendon architecture in humans remains limited. The leukocyte content of PRP preparations is likely to influence biological activity: leukocyte-poor PRP (LP-PRP) is generally favored for intra-articular applications, whereas leukocyte-rich PRP (LR-PRP) has been proposed for some recalcitrant tendinopathies; however, optimal formulation remains unsettled (8). Mechanical loading may complement PRP by activating overlapping repair pathways, providing a rationale for combining injection therapy with structured rehabilitation rather than using PRP as a stand-alone intervention (9, 10).

Despite the substantial growth in PRP research, clinical practice remains inconsistent, partly reflecting heterogeneity in preparation methodology, injection protocols, and patient selection criteria. Critically appraising the available evidence and translating it into actionable clinical guidance is therefore of considerable practical importance.

The present narrative review aims to synthesize the current clinical evidence for PRP in the management of degenerative joint disorders and soft tissue conditions, to evaluate indication-specific efficacy against relevant comparators, and to provide evidence-informed recommendations for its use in musculoskeletal practice.

## 2 Methods

### 2.1 Literature search strategy

As this is a narrative—rather than a systematic—review, no protocol was registered and no PRISMA-compliant flow diagram was generated. Relevant literature was identified through searches of the electronic databases Medline (via PubMed), Embase, and the Cochrane Database of Systematic Reviews for publications available up to July 2025. The search strategy combined Medical Subject Headings (MeSH) and free-text terms, including: “platelet-rich plasma”, “PRP”, “platelet concentrate”, “osteoarthritis”, “knee osteoarthritis”, “hip osteoarthritis”, “tendinopathy”, “lateral epicondylitis”, “tennis elbow”, “plantar fasciitis”, “rotator cuff tendinopathy”, “Achilles tendinopathy”, “patellar tendinopathy”, “trochanteric tendinopathy”, “musculoskeletal”, “intra-articular injection”, and “regenerative medicine”, linked with the Boolean operators AND/OR. Results were restricted to human studies published in English or a Scandinavian language (Danish, Norwegian, or Swedish), and reference lists of key publications were hand-searched to identify additional relevant sources.

### 2.2 Inclusion and exclusion criteria

Priority was given to publications that investigated PRP as an active intervention for the treatment of osteoarthritis or tendinopathy in adult populations, compared PRP against an active comparator (e.g., corticosteroid, hyaluronic acid, physiotherapy) or placebo, and reported outcomes relevant to clinical practice—including validated pain scales [e.g., Visual Analog Scale (VAS), Numerical Rating Scale (NRS)], functional scoring instruments [e.g., Western Ontario and McMaster Universities Osteoarthritis Index (WOMAC), Harris Hip Score, American Orthopedic Foot and Ankle Society (AOFAS) score], or structural outcomes assessed by imaging. Emphasis was placed on randomized controlled trials (RCTs), systematic reviews, and meta-analyses, supplemented by influential consensus statements and clinical practice guidelines where these informed contemporary clinical reasoning. Case series, animal studies, and studies with a sample size of fewer than ten participants were generally not relied upon, although exceptions were made where they provided unique mechanistic or longitudinal context.

### 2.3 Study selection and data synthesis

Titles, abstracts, and full texts were reviewed by the authors and selected for inclusion on the basis of clinical relevance, methodological quality, and recency, rather than through dual independent screening or a pre-specified protocol. Screening and study selection were undertaken by the authors, with any disagreements resolved by discussion and consensus. No standardized risk-of-bias assessment was applied; instead, the certainty of evidence for each indication is described qualitatively in the relevant results sections, drawing where possible on previously published assessments such as GRADE ratings reported within referenced systematic reviews. Given the substantial heterogeneity in PRP preparation, injection protocols, and outcome measures across the literature, no meta-analytic pooling of effect sizes was performed. Findings were synthesized narratively and organized by anatomical region and clinical indication. Where multiple meta-analyses were available for a given indication, the most recent and methodologically rigorous were emphasized. Clinical recommendations were formulated on the basis of this synthesis, taking into account effect size, durability of benefit, safety profile, and the practical requirements of clinical implementation, and should therefore be regarded as interpretive rather than the product of a formal evidence-grading process. Consistent with a narrative rather than a systematic approach, formal counts of records retrieved and screened were not maintained; in addition to database searching, relevant publications were also identified pragmatically from the reference lists of key articles, recent clinical reviews, and consensus documents.

## 3 Results

Table 1 summarizes the pivotal evidence, comparators, sample sizes, between-group effect sizes with 95% confidence intervals, clinical importance, and certainty for each indication discussed below.

**Table 1:** Summary of pivotal evidence for platelet-rich plasma by indication.

Indication	Pivotal evidence (type)	Comparator	*n*	Between-group effect (95% CI), time point	Clinical importance/MCID	Certainty	Conclusion
Knee OA	Tan 2021 (meta, 26 RCTs); Di Martino 2019 (RCT)	Hyaluronic acid	2,430 (Tan); 192 (RCT)	WOMAC total MD −5.04 (−8.82 to −1.26) at 3 mo; −8.52 (−11.17 to −5.87) at 6 mo; −10.52 (−13.77 to −7.27) at 12 mo (favors PRP). RCT: no between-group superiority vs. HA; reintervention 22.6% vs. 37.1% at 24 mo *(P* = 0.036)	6- and 12-mo WOMAC exceed MCID (6 pts)	Moderate (Level I meta); high heterogeneity	Modest superiority over HA; not superior on all measures
Hip OA	Lim 2023 (meta, 8 studies)	Baseline (largely uncontrolled)	331	VAS MD −1.6 (−2.3 to −0.9) at end-point vs. baseline; function significant only at 1–2 mo	Near/below MCID (1.5–2.0) → modest	Low–moderate; no true control arm	Modest, uncertain average effect
Ankle OA	Paget 2021 (RCT, sham)	Saline placebo	100	AOFAS adjusted between-group diff −1 (−6 to 3) over 26 wk (ns); sensitivity −2 (−8 to 3)	No clinically important benefit	Moderate–high (blinded RCT)	Not supported
Lateral elbow	Karjalainen 2021 (Cochrane, placebo); Mishra 2014 (RCT, active)	Placebo; active control	2,337 (Cochrane); 230 (Mishra)	Cochrane: little/no clinically important benefit vs. placebo at 3 mo (GRADE moderate). Mishra: pain improvement 71.5% vs. 56.1% at 24 wk (*P* = 0.019); 55.1% vs. 47.4% at 12 wk (ns)	Not important vs. placebo	Moderate (Cochrane)	Investigational; not routine — placebo evidence negative
Plantar fasciopathy	Hohmann 2021 (meta)	Corticosteroid	15 studies	VAS SMD −0.84 (−1.02 to −0.67) at 3 mo; −1.98 (−3.23 to −0.74) at 6 mo; −1.71 (−3.13 to −0.28) at 12 mo (favors PRP). AOFAS SMD −2.51 (−4.40 to −0.62) at 6 mo; −2.73 (−4.78 to −0.67) at 12 mo	Favors PRP from 3 mo	Low–moderate; I^2^≈95%, high RoB → caution	Superior to corticosteroid at 3–12 mo; placebo evidence lacking
Rotator cuff/subacromial	Rossi 2024 (RCT)	Corticosteroid	100	12-mo ASES 89.3 vs. 78.0; SANE 89.2 vs. 80.5; VAS 1.68 vs. 2.3 (all *P* < 0.001); treatment failure 12% vs. 30% (*P* < 0.001). (No. 95% CI in abstract.)	Favors PRP at 6 & 12 mo	Low–moderate; single RCT (abstract)	Benefit vs. corticosteroid; placebo-controlled efficacy not established
Gluteal/trochanteric	Fitzpatrick 2019 (RCT + open-label ext.)	Corticosteroid	80	mHHS 77.6 vs. 65.7 at 24 wk (*P* = 0.0003); 74.1 vs. 67.1 at 12 wk (*P* = 0.048). 27 corticosteroid non-responders crossed over to PRP	Favors PRP	Low–moderate; open-label beyond 12 wk; crossover	Favorable in recalcitrant disease; long-term data open-label
Patellar	Scott 2019 (RCT, saline)	Saline (+ eccentric loading)	57	No significant between-group difference in VISA-P at any time point; 12-mo VISA-P 58 (LR-PRP)/71 (LP-PRP)/80 (saline), *P* > 0.05	Not demonstrated	Moderate (blinded RCT)	Not established; caution in refractory disease
Achilles (midportion)	Kearney 2021 (RCT, sham)	Sham (dry needle)	240	VISA-A adjusted MD −2.7 (−8.8 to 3.3) at 6 mo (primary; ns); −0.4 (−5.1 to 4.3) at 3 mo	Far below MCID (12 pts)	High (blinded RCT)	Not recommended

MD, mean difference; SMD, standardized mean difference; CI, confidence interval; MCID, minimum clinically important difference; HA, hyaluronic acid; RoB, risk of bias; WOMAC, Western Ontario and McMaster Universities Osteoarthritis Index; AOFAS, American Orthopedic Foot & Ankle Society; ASES, American Shoulder and Elbow Surgeons; SANE, Single Assessment Numeric Evaluation; VISA-A/-P, Victorian Institute of Sport Assessment–Achilles/–Patella; mHHS, modified Harris Hip Score; ns, not significant.

### 3.1 Mechanism of action and biological rationale

PRP is prepared by centrifugation of autologous whole blood, yielding a plasma fraction enriched in platelets. Historically, PRP has been described in relative terms—typically four to seven times greater than baseline platelet concentration—but more recent consensus emphasizes the absolute platelet dose delivered. A working target of approximately 10 × 10^9^ (10 billion) platelets per treatment has been proposed as an emerging reference target rather than an established threshold; whether sub-threshold dosing accounts for some null findings remains unproven in the absence of direct dose–response data (11). Upon injection into the target tissue, activated platelets degranulate and release a cascade of growth factors and bioactive proteins that are proposed to modulate inflammation, stimulate cellular proliferation, and influence extracellular matrix turnover (1, 4).

Proposed mechanisms for the clinical effect of PRP differ between articular and tendinous tissue. In articular cartilage and synovium, PRP is thought to modulate catabolic cytokines—including IL-1β and TNF-α–and to stimulate chondrocyte proliferation and matrix synthesis, supporting a putative shift toward an anabolic environment (1, 5). In tendinous tissue, TGF-β and PDGF are implicated in tenocyte proliferation and collagen type I synthesis, whilst VEGF has been associated with neovascularisation of poorly perfused tendinopathic regions (5). These tissue-specific observations provide a biological rationale for tailoring PRP formulation to the target indication—with LP-PRP generally favored for intra-articular use and LR-PRP proposed for some tendinopathic applications—but this distinction should not be interpreted as settled clinical doctrine (8). It should be emphasized, however, that this mechanistic framework is largely derived from *in vitro* and animal data. Although some studies have demonstrated disease-modifying effects in humans, the extent to which these proposed pathways translate into clinically meaningful tissue repair remains insufficiently explored, and the optimal formulation across indications has yet to be definitively established—as reflected in current consensus statements (12).

The therapeutic response to PRP, where present, is characteristically delayed relative to corticosteroid injection, with clinical improvement often emerging gradually over 4–12 weeks and peak effect reported around 12–16 weeks in some studies. The duration of benefit may be longer than corticosteroid injection in selected indications—extending to 18 months in some articular studies and up to 24 months in selected tendinopathy cohorts (4, 13). This temporal profile has important implications for patient counseling and outcome assessment, although prolonged follow-up cannot compensate for inadequate placebo control or poor study design.

### 3.2 Degenerative joint disorders

#### 3.2.1 Knee osteoarthritis

Knee osteoarthritis (KOA) represents the most prevalent degenerative joint disorder of the lower limbs and the most extensively studied indication for PRP in the articular context. The available evidence—comprising numerous RCTs and several meta-analyses—suggests that PRP may produce somewhat more durable symptomatic improvement than corticosteroids and modestly superior outcomes to hyaluronic acid (HA) in selected patients with mild-to-moderate disease. Effect sizes are generally small to moderate and vary considerably by disease severity, comparator, PRP formulation, injection protocol, and study quality; placebo-controlled data are less abundant than active-comparator data and yield more modest estimates of intrinsic treatment effect.

In active-comparator trials of PRP against intra-articular corticosteroid, a recurring observation is a time-dependent reversal of effect: corticosteroid provides faster early symptom relief, but PRP-treated patients tend to show comparable or superior outcomes from approximately 3 months onward, with the intergroup difference widening at 6 and 12 months. This temporal pattern is consistent with the recognized decline in corticosteroid efficacy over the medium term—and possible adverse effects on cartilage with repeat use—rather than with a uniformly strong intrinsic effect of PRP (1). In a double-blind RCT by Di Martino et al. (2) [*n* = 192; Kellgren–Lawrence (KL) grade 0–3], both PRP and hyaluronic acid (HA) produced significant clinical improvements that were maintained over a mean follow-up of 64 months; at final evaluation, only the PRP group retained values significantly higher than baseline. Notably, the rate of reintervention at 24 months was significantly lower in PRP-treated patients (22.6% vs. 37.1%, *P* = 0.036), suggesting greater durability of clinical benefit despite comparable mean functional scores between groups at individual follow-up points. The meta-analyses of Tan et al. (14) (26 RCTs; *n* = 2,430) and Dai et al. (15) have likewise reported superiority of PRP over HA on WOMAC and VAS outcomes at medium-term follow-up (in the meta-analysis of Tan et al., the WOMAC total mean difference favored PRP by −8.52 points [95% CI −11.17 to −5.87] at 6 months and −10.52 points [95% CI −13.77 to −7.27] at 12 months, exceeding the approximate six-point minimal clinically important difference), although heterogeneity remains substantial and placebo-controlled effect estimates are smaller.

The dose-response relationship for injection frequency has also been explored. Zhuang et al. (16) demonstrated that both three and five injections produced superior outcomes compared with a single injection, yet without significant difference between the three- and five-injection regimens, suggesting a possible saturation threshold. This is corroborated by Tao et al. (17), whose meta-analysis reported superiority of three injections over one. The available comparative studies mainly contrast one vs. three (or one, three, and five) injections and do not establish a universal protocol; two to three injections at one-to-three-week intervals are commonly used, but the interval, the timing of peak benefit, and durations such as 18 months should be regarded as indicative rather than firmly established (16, 18).

Beyond symptomatic relief, exploratory investigations have examined imaging surrogates of structural response. MRI-based studies report reduced cartilage volume loss and better preservation of cartilage thickness in PRP-treated patients relative to controls (10, 14, 19), whilst ultrasound studies document concurrent reductions in synovial inflammation and joint effusion (20). These observations are hypothesis-generating: symptom durability and improvements in imaging surrogates do not, on their own, establish disease-modifying efficacy. Demonstration of disease modification in osteoarthritis would require evidence of slowed radiographic progression, preserved cartilage morphology or composition, or delayed arthroplasty in adequately powered trials with pre-specified structural endpoints — none of which have yet been achieved for PRP.

#### 3.2.2 Hip osteoarthritis

Hip osteoarthritis (HOA) presents greater technical and biological complexity than KOA, reflecting the deeper articular location, the substantial capsular and muscular envelope, and the frequent co-occurrence of subchondral remodeling and cam or pincer morphology (21–24). The available evidence suggests that PRP may provide symptomatic benefit in selected patients with HOA, but the evidence base is smaller and less definitive than for KOA.

Dallari et al. (25) conducted a three-arm randomized study comparing ultrasound-guided, double-centrifuged LP-PRP, HA, and the combination of PRP plus HA, administered as three weekly injections (PRP 5 ml per injection). This trial did not include a saline or other inert placebo arm and therefore does not provide placebo-controlled evidence for hip OA. The PRP group showed better pain and functional outcomes (WOMAC, VAS, Harris Hip Score) than the comparators, with the most pronounced differences at the earlier (two- and six-month) time points; between-group differences were not uniformly significant for every outcome at every time point, and PRP did not demonstrate a consistent advantage over the PRP-plus-HA combination. As the study was not double-blinded, these findings should be interpreted with corresponding caution. Battaglia et al. (26) reported comparable findings with two injections at a 2-week interval. The meta-analysis of Lim et al. (27) (8 studies; *n* = 331 patients) demonstrated a statistically significant reduction in VAS pain at end-point follow-up relative to baseline (mean difference −1.6; 95% CI −2.3 to −0.9), with a significant functional improvement seen only at early 1–2-month follow-up. Because the hip trials largely lack a true control arm, this represents an uncontrolled within-group reduction rather than a placebo-controlled treatment effect; its magnitude is close to common minimum clinically important difference estimates (typically 1.5–2.0) and should accordingly be regarded as modest and uncertain. Overall, the hip literature supports cautious consideration of PRP in selected patients, but conclusions are limited by fewer trials, smaller sample sizes, the dominance of active-comparator over placebo-controlled designs, and technical variability in injection technique and preparation protocols.

As in KOA, commonly used protocols comprise two to three injections at 1–3-week intervals, with peak response around 12 weeks and clinically meaningful improvement reported for 12–18 months in some studies (8, 22). Administration of more than three injections does not appear to confer additional benefit in knee OA data, suggesting a possible saturation effect, although direct extrapolation to hip OA should be cautious (28). LP-PRP formulations and a total injectate volume of < 15 ml have been associated with more favorable outcomes in available hip studies (22). Given the depth of the hip joint, injection should be performed under ultrasound or fluoroscopic guidance to ensure reliable intra-articular placement.

#### 3.2.3 Other joint osteoarthritis

Although the preponderance of evidence pertains to the knee and hip, clinical investigations have also been conducted in the shoulder, ankle, acromioclavicular (AC), and carpometacarpal (CMC) joints (27, 29–39). Several of these articulations tend to have smaller joint volumes and thinner articular cartilage than the large weight-bearing joints, although this varies between them; such differences may influence both the biological response to PRP and the technical requirements of injection. The evidence base in these joints is limited and uneven. Some trials report symptomatic improvement, predominantly in shoulder osteoarthritis and in the frozen-shoulder literature; adhesive capsulitis (frozen shoulder) is, however, a distinct entity from osteoarthritis and is referred to here only for contextual completeness. By contrast, ankle osteoarthritis is the indication with the strongest negative placebo-controlled signal: in a double-blind randomized trial, Paget et al. (32) found no clinically meaningful benefit of PRP over saline placebo on ankle pain or function at 26 weeks (adjusted between-group difference in AOFAS score −1 point [95% CI −6 to 3]), and the open-label extension study (33) did not establish placebo-controlled efficacy. The systematic reviews by Vannabouathong et al. (35) and Laohajaroensombat et al. (34) draw predominantly on open-label or low-quality comparative data and should be interpreted in light of this negative high-quality placebo-controlled evidence. Robust conclusions regarding optimal protocol, formulation, and long-term outcomes in these joints—and clarification of whether PRP has any role at all in ankle osteoarthritis—await the completion of further adequately powered placebo-controlled trials.

### 3.3 Soft tissue disorders

#### 3.3.1 Lateral epicondylitis

Lateral epicondylitis (LE), or tennis elbow, is characterized by degeneration of the extensor carpi radialis brevis tendon at its humeral origin. It is one of the most extensively investigated indications for PRP in tendinopathy, but the evidence base is conflicting, and a clear distinction must be drawn between trials comparing PRP with corticosteroid and those comparing PRP with placebo, since these comparisons yield very different conclusions (40, 41).

Against corticosteroid, PRP shows a characteristic time-dependent advantage: corticosteroid provides more rapid early relief, but PRP-treated patients consistently report superior pain and function at 6–12 months, in keeping with the recognized decline in corticosteroid efficacy and concerns regarding its possible adverse effects on tendon quality with repeat use (40). In a double-blind, multicentre RCT by Mishra et al. (40) (*n* = 230), PRP-treated patients achieved a 71.5% pain reduction at 24 weeks compared with 56.1% in the active control group (*P* = 0.019), with 83.9% attaining a clinically meaningful response. Sahbaz et al. (42) compared PRP with extracorporeal shockwave therapy (ESWT) in chronic lateral epicondylitis and reported significantly greater improvement with PRP (*P* < 0.001). Long-term retrospective data from Hastie et al. (43) reported that 87% of participants experienced significant functional improvement and observed a low rate of subsequent surgery; as an uncontrolled retrospective series, however, this design cannot establish that PRP reduces the need for surgery and is subject to selection and attrition bias. Against placebo, however, the picture is markedly less favorable. The 2021 Cochrane systematic review by Karjalainen et al. (41) (32 RCTs, *n* = 2,337) concluded that autologous blood or PRP probably provides little or no clinically important benefit in pain or function compared with placebo injection at 3 months (moderate-certainty evidence), with no clinically important benefit observed at 6–12 months either. The reviewers concluded that, in the absence of evidence of benefit, the costs and risks of these injections are not justified. The apparent superiority of PRP in many head-to-head trials may therefore reflect, at least in part, the underperformance of corticosteroid in the medium term rather than a strong intrinsic effect of PRP—a nuance with direct implications for transparent patient counseling.

Most positive comparator trials in lateral epicondylitis have employed a single injection of 2–4 ml of LR-PRP into the extensor communis enthesis, often delivered via fenestration technique under real-time ultrasound guidance. One to two injections at 2–4-week intervals have been used in longstanding tendinopathy exceeding 6 months, with reported improvement emerging at 6–12 weeks and persisting for 12–24 months in some cohorts (40, 42). Given the negative placebo-controlled evidence, however, this protocol should be presented as investigational or selectively considered rather than as established standard care.

#### 3.3.2 Plantar fasciitis

Plantar fasciitis (PF), characterized by degeneration of the proximal plantar fascial insertion at the calcaneal tuberosity, is among the most prevalent causes of heel pain. PRP is one of the more frequently studied soft tissue indications for PRP, but—as in lateral epicondylitis—the conclusions depend heavily on the comparator. Active-comparator trials vs. corticosteroid have been broadly favorable to PRP in the medium term, whereas placebo-controlled data remain sparse and provide a less definitive picture of intrinsic treatment effect.

Corticosteroids remain effective at 2–6 weeks but carry recognized risks of plantar fat pad atrophy and fascial rupture with repeated use (28). In the meta-analysis by Hohmann et al. (28), which incorporated 18 RCTs, PRP demonstrated significantly superior outcomes compared with corticosteroid at 6–12 months across both VAS pain and functional scores (AOFAS, Foot and Ankle Disability Index [FADI]); in that meta-analysis the VAS standardized mean difference favored PRP by −1.98 [95% CI −3.23 to −0.74] at 6 months and −1.71 [95% CI −3.13 to −0.28] at 12 months, although high statistical heterogeneity (*I*^2^ ≈ 95%) and risk of bias in the included studies warrant caution. Zuo et al. (8) and Desouza and Shetty (44) reported concordant findings, with meaningful between-group differences emerging from 3 months and persisting through 12 months of follow-up. PRP has also been associated in some ultrasound studies with reductions in plantar fascial thickness and improvements in echogenicity and vascular profile; these findings are compatible with structural change but should not be equated with proven tissue regeneration (45). As with lateral epicondylitis, however, the apparent superiority of PRP over corticosteroid at 6–12 months may reflect, in part, the loss of corticosteroid efficacy beyond the early weeks rather than a strong intrinsic effect of PRP. The placebo-controlled evidence base in plantar fasciitis is comparatively limited and an unequivocal demonstration of intrinsic efficacy vs. placebo remains outstanding.

Most RCTs have used a single injection of 2–4 mL of PRP—frequently, though not consistently, characterized as leukocyte-poor—into the proximal fascial insertion under ultrasound guidance. Symptomatic improvement typically emerges over 6–12 weeks, with sustained benefit for up to 12 months (8, 28).

#### 3.3.3 Rotator cuff tendinopathy and subacromial pain syndrome

Rotator cuff tendinopathy and subacromial pain syndrome collectively represent the most common cause of shoulder pain in adults, but they encompass several distinct entities—degenerative rotator cuff tendinopathy, partial-thickness and full-thickness tears, subacromial (bursal) pain syndrome, and the post-surgical augmentation setting—that differ in pathology, injection target (subacromial or bursal vs. intratendinous), and evidence base, and should not be pooled as a single condition. Most controlled data concern PRP vs. corticosteroid or physiotherapy in rotator cuff tendinopathy and subacromial pain syndrome, where the therapeutic profile appears to mirror that of other tendinopathies: minimal short-term advantage over corticosteroid (< 4 weeks), followed in some studies by progressive improvement from 2–12 months compared with corticosteroid injection or physiotherapy alone (31, 39, 46–48). Injection targets vary across these trials—most commonly subacromial or bursal placement rather than intratendinous injection—which further limits pooling. A double-blind RCT by Rossi et al. (48) demonstrated that subacromial PRP injection produced significantly greater improvements in pain and functional outcomes compared with corticosteroid at 12 months (for example, ASES 89.3 vs. 78.0 and VAS 1.68 vs. 2.3 at 12 months, both *P* < 0.001, with treatment failure in 12% vs. 30%), with the treatment difference continuing to widen over the follow-up period; confidence intervals were not reported in the available abstract. As throughout this review, however, superiority over corticosteroid does not establish placebo-controlled efficacy: high-quality, blinded, sham-controlled trials of subacromial or rotator cuff PRP are scarce, so the intrinsic treatment effect remains uncertain. The impression that benefit is most consistent in degenerative, partial-thickness disease rests on indirect and heterogeneous data rather than a direct comparative synthesis with effect estimates, and should be regarded as tentative; evidence for full-thickness tears and for post-surgical augmentation is limited and inconclusive and should be considered separately. Editorial commentaries should not be relied upon as evidence where primary trials or systematic reviews are available.

#### 3.3.4 Trochanteric and gluteal tendinopathy

Gluteus medius and minimus tendinopathy, the primary substrate of greater trochanteric pain syndrome, may respond favorably to PRP, particularly in patients with recalcitrant, longstanding disease. In a double-blind RCT by Fitzpatrick et al. (13) (*n* = 80), a single LR-PRP injection produced significantly greater improvement than corticosteroid at the primary 12-week endpoint (modified Harris Hip Score 74.1 vs. 67.1; *P* = 0.048), with a larger between-group difference at 24 weeks (77.6 vs. 65.7; *P* = 0.0003). Longer-term outcomes were assessed in an open-label extension in which patients with an unsatisfactory response could receive PRP after the randomized phase; the reported 12- and 24-month results therefore reflect a longer-term, partly open-label cohort rather than a maintained blinded head-to-head comparison. Among patients followed to 2 years, approximately 80% maintained a clinically meaningful improvement, although the denominator and any crossover should be interpreted with caution. Therapeutic effect typically emerges at 8–12 weeks, with durability extending to 24 months in this trial (13).

#### 3.3.5 Patellar tendinopathy

For patellar tendinopathy (“jumper's knee”), the evidence base is heterogeneous and methodologically uneven, with small sample sizes, variable blinding, and inconsistent PRP formulations limiting the strength of inference that can be drawn. Active-comparator and observational data have suggested that multiple PRP injections may produce better outcomes than a single administration, and that any benefit appears most evident in patients with moderate-to-severe, refractory tendinopathy (49–51). The most rigorous placebo-controlled evidence, however, does not corroborate a strong intrinsic effect. In the double-blind RCT by Scott et al. (52) (*n* = 57), patients with chronic patellar tendinopathy were randomized to LR-PRP, LP-PRP, or saline injection alongside eccentric loading exercises; no significant difference between any of the three groups was observed at 12 weeks, 6 months, or 12 months on the VISA-P score (at 1 year, mean VISA-P was 58 for LR-PRP, 71 for LP-PRP, and 80 for saline; *P* > 0.05 for all). Earlier positive trials such as Dragoo et al. (50) used dry-needling as the comparator rather than placebo, which complicates interpretation. Taken together, the data permit only cautious consideration of PRP for refractory patellar tendinopathy that has not responded to a structured eccentric or heavy slow resistance loading programme; firm recommendations cannot yet be made, and further adequately powered placebo-controlled RCTs are required.

#### 3.3.6 Achilles tendinopathy

In contrast to the indications discussed above, PRP is not recommended for the management of Achilles tendinopathy. Multiple RCTs, systematic reviews, and meta-analyses consistently demonstrate no clinically or statistically significant difference in pain, function, or structural outcomes compared with placebo or structured rehabilitation (6, 7, 53–56). A high-quality RCT by Kearney et al. (55) (*n* = 240) found no benefit of PRP over sham injection in chronic midportion Achilles tendinopathy at its primary 6-month endpoint (adjusted mean difference in VISA-A score −2.7 [95% CI −8.8 to 3.3], well within the 12-point minimal clinically important difference), with no significant difference at the 3-month time point either (mean difference −0.4 [95% CI −5.1 to 4.3]). The reasons for this apparent lack of efficacy are not fully established but may relate to the specific biological milieu of the Achilles tendon, the natural history of the condition, or the concurrent use of structured loading programmes as the standard of care comparator in most trials. Notably, the pivotal sham-controlled evidence concerns chronic midportion Achilles tendinopathy; extrapolation to insertional Achilles tendinopathy, where the evidence base differs, should be made with caution. Based on current evidence, PRP cannot be recommended for Achilles tendinopathy and should not be offered as an alternative to evidence-based rehabilitation.

## 4 Discussion

### 4.1 Synthesis of findings

The present review suggests that PRP may have a clinically meaningful role in selected musculoskeletal indications, particularly mild-to-moderate knee and hip osteoarthritis, plantar fasciitis, degenerative rotator cuff tendinopathy, and trochanteric tendinopathy. However, the strength of evidence is highly indication-dependent and varies according to comparator, PRP formulation, injection protocol, and follow-up duration. In lateral epicondylitis, apparent superiority over corticosteroid must be interpreted cautiously because placebo-controlled evidence does not confirm a clinically important intrinsic treatment effect.

A recurring theme across indications with supportive evidence is the delayed time course of PRP response. The delayed onset—typically 8–16 weeks—may reflect slower biological processes of cellular signaling, matrix turnover, and tissue remodeling, but clinical improvement cannot be assumed to represent structural repair. This characteristic has implications for clinical trial design and patient counseling: very short follow-up may underestimate delayed symptomatic effects, whereas inadequate placebo control may overestimate true treatment efficacy.

### 4.2 Methodological limitations in the literature

Despite the breadth and growing volume of PRP research, the field is burdened by substantial methodological heterogeneity that complicates interpretation and limits the generalisability of individual study findings. A fundamental challenge is that PRP is not a homogeneous therapeutic product: centrifugation protocol, blood volume, anticoagulant use, activation method, and leukocyte content each independently influence the final composition and biological activity of the preparation, yet the majority of published trials provide insufficient detail on these parameters (12). This renders cross-study comparisons unreliable and obscures the relationship between formulation characteristics and clinical outcomes.

Null results may reflect true lack of efficacy in some indications, but may also be influenced by methodological factors such as advanced disease stage, inappropriate formulation, insufficient dosing, inadequate rehabilitation protocols, or limited follow-up. Frequently identified sources of bias and confounding include the enrolment of patients with end-stage disease (KL grade IV osteoarthritis), the use of an inappropriate PRP formulation for the target tissue, and the absence of a structured post-injection rehabilitation programme (48, 57). Inadequate or absent blinding, small and heterogeneous study populations with insufficient statistical power, and the use of active comparators of varying biological activity further limit interpretability (1, 58, 59). Inconsistent reporting of platelet concentration, leukocyte content, and activation status across publications also impedes comparison and meta-analytic synthesis.

### 4.3 Clinical implications and contraindications

Professional guidelines and consensus statements do not take a uniform position on PRP in osteoarthritis (Table 2). Rheumatology-focused guidance is cautious or negative—the OARSI guidelines do not recommend PRP (60), and the ACR/Arthritis Foundation guideline issues a strong recommendation against its use in knee and hip osteoarthritis (61)—whereas sports-medicine and orthobiologic consensus documents (AMSSM; ESSKA-ORBIT) (1, 12) are more permissive, endorsing responsible, transparent use in selected patients while emphasizing standardization and higher-quality evidence. This discordance should be borne in mind when interpreting the indication-specific suggestions below, which are offered as pragmatic guidance rather than formal graded recommendations.

**Table 2:** Positions of major professional organizations and consensus groups on PRP in osteoarthritis.

Organization (Reference)	Scope	Position on PRP in OA	Basis/key qualifier
OARSI 2019 (60)	Knee, hip, polyarticular OA	Not recommended	Uncertainty in PRP product characterization; heterogeneous evidence
ACR/AF 2019 (61)	Hand, hip, knee OA	Strong recommendation against	Lack of standardization of preparations; heterogeneous trial evidence
AMSSM 2021 (1)	Sports medicine (regenerative)	Responsible, transparent use; no blanket endorsement of routine use	Emphasizes informed consent, standardized reporting, shared decision-making, and higher-quality research
ESSKA-ORBIT 2024 (12)	Knee OA (blood-derived orthobiologics)	Considered an option in selected patients (conditional/positive)	Calls for standardized preparation and reporting; leukocyte-poor PRP generally favored intra-articularly
EULAR 2021 (3)	Intra-articular therapies (general)	No specific recommendation for PRP; cautious	Procedural/safety guidance; evidence for PRP regarded as insufficient for a specific recommendation

PRP, platelet-rich plasma; OA, osteoarthritis; OARSI, Osteoarthritis Research Society International; ACR/AF, American College of Rheumatology/Arthritis Foundation; AMSSM, American Medical Society for Sports Medicine; ESSKA-ORBIT, European Society for Sports Traumatology, Knee Surgery and Arthroscopy – Orthobiologic Initiative; EULAR, European Alliance of Associations for Rheumatology.

The evidence reviewed herein supports a cautious and indication-specific clinical framework for PRP use in musculoskeletal medicine. PRP may be considered as a second-line intervention in selected patients with mild-to-moderate knee or hip osteoarthritis, plantar fasciitis, degenerative rotator cuff tendinopathy, or trochanteric tendinopathy after failure of structured conservative management. It should not be offered as an alternative to evidence-based rehabilitation for Achilles tendinopathy, and its use in lateral epicondylitis should be framed cautiously given negative placebo-controlled evidence. The role of PRP in ankle osteoarthritis remains unsupported by high-quality placebo-controlled data.

Clinical outcomes appear to depend on several factors: LP-PRP is generally favored for intra-articular applications, whilst LR-PRP has been proposed for some tendinopathies; injection number and interval should be indication-specific rather than uniform; and post-injection rehabilitation should be structured and progressive. The integration of mechanical loading with PRP may be biologically plausible because both stimuli activate overlapping repair pathways, but the magnitude of any synergistic clinical effect remains uncertain (9, 10).

PRP is contraindicated in the presence of local or systemic infection and should generally be avoided or deferred in patients with active or recently diagnosed malignant disease, depending on oncological context and local policy. Some clinicians defer PRP in patients who have recently received systemic corticosteroids or NSAIDs, on the theoretical basis that these agents may impair platelet function; however, the evidence for specific withholding intervals—such as 2–4 weeks for corticosteroids or 48 h for NSAIDs—is limited and largely consensus-based, and is likely to vary with the agent, dose, and half-life. Mixing local anesthetic agents directly into the injectate is often avoided because of largely preclinical concerns that they may reduce platelet and tenocyte viability; this is distinct from the use of superficial local anesthesia for skin analgesia, which is not implicated (1, 41). In the absence of these contraindications, PRP has a favorable overall safety profile; adverse effects—predominantly local tenderness, transient swelling, or a brief post-injection pain flare—are generally mild, predictable, and self-limiting, with the Cochrane review by Karjalainen et al. (41) reporting adverse event rates of approximately 17%−19% across pooled lateral elbow trials, comparable to placebo injection (low-certainty evidence); indication-specific safety data for other musculoskeletal applications remain more limited (1, 41). A further consideration is cost. PRP is not reimbursed in most European public-health settings and is typically delivered as an out-of-pocket intervention. Direct costs vary substantially by country and center, and multi-injection protocols accordingly multiply the cumulative expense. Where the intrinsic efficacy of PRP over placebo is uncertain—as is currently the case for lateral epicondylitis, ankle osteoarthritis, and Achilles and patellar tendinopathy—the cost-benefit balance is correspondingly weak, and patients should be counseled accordingly. This consideration aligns with the AMSSM Position Statement (1), which emphasizes the importance of transparent communication regarding the evidentiary basis, cost, and clinical alternatives for any regenerative medicine intervention.

### 4.4 Directions for future research

Progress in the field requires a concerted effort to address the methodological inconsistencies that have characterized PRP research to date. Standardization of PRP preparation and characterization—including mandatory reporting of platelet concentration, leukocyte and red blood cell content, and activation protocol—is an essential prerequisite for meaningful inter-study comparison (11, 12). Future RCTs should incorporate extended follow-up periods of at least 12 months as a minimum, use validated and indication-specific patient-reported outcome measures, and adopt placebo-controlled designs where the intrinsic effect of PRP remains uncertain, alongside active comparators where clinically appropriate. Head-to-head comparisons of LP-PRP and LR-PRP across both articular and tendinous indications would provide valuable guidance on formulation selection. Whether PRP exerts any disease-modifying effect in osteoarthritis remains unknown; current imaging and ultrasound observations are hypothesis-generating only. Definitive trials would require pre-specified structural endpoints (radiographic progression, MRI cartilage morphology, T2/T1ρ relaxometry) and clinically relevant hard endpoints such as time to arthroplasty, in adequately powered, long-term, placebo-controlled designs.

## 5 Conclusion

PRP may be a reasonable second-line option for selected patients with mild-to-moderate knee or hip osteoarthritis, plantar fasciitis, trochanteric tendinopathy, and degenerative rotator cuff tendinopathy, particularly after failure of structured conservative care. The strongest clinical rationale exists in scenarios where PRP's medium- and long-term effect exceeds that of corticosteroid injections; however, the certainty of evidence varies substantially by indication, comparator, formulation, and follow-up duration. Current evidence does not support PRP for Achilles tendinopathy. Placebo-controlled data challenge its use in lateral epicondylitis—where benefits over corticosteroid in the medium term are not replicated against placebo—and in ankle osteoarthritis, where a high-quality placebo-controlled RCT was negative. Claims of tissue regeneration or disease modification in osteoarthritis, often inferred from imaging surrogates, remain insufficiently established and should be made with caution.

Where PRP is used, outcomes appear to depend on appropriate patient selection, formulation choice (with LP-PRP generally favored for articular applications and LR-PRP proposed for selected tendinous conditions), adherence to multi-injection protocols where indicated, and integration with structured active rehabilitation. The heterogeneity of the existing literature—attributable in large part to inconsistent preparation methodology, comparator choice, and follow-up duration—limits the strength of indication-specific recommendations. Standardized PRP characterization (with mandatory reporting of platelet, leukocyte, and red-cell content and activation protocol), rigorous placebo-controlled trials in indications where placebo data are weak or absent, and long-term studies incorporating structural as well as symptomatic endpoints are needed before stronger recommendations can be supported.
